# What influences individual preferences for responsiveness in oral health services? A discrete choice experiment in Türkiye

**DOI:** 10.1136/bmjopen-2025-106411

**Published:** 2025-11-21

**Authors:** Murat Özbek, Mahmut S Yardım

**Affiliations:** 1 Zonguldak Provincial Health Directorate, Zonguldak, Türkiye; 2 Graduate School of Health Sciences, PhD Program in Health Management, Üsküdar University, İstanbul, Türkiye; 3 Faculty of Medicine, Department of Public Health, Hacettepe University, Ankara, Türkiye

**Keywords:** Patient Preference, PUBLIC HEALTH, Health Services, HEALTH ECONOMICS, Delivery of Health Care, Integrated, ORAL MEDICINE

## Abstract

**Objectives:**

This study aims to evaluate individual preferences that influence responsiveness in oral healthcare services through a discrete choice experiment (DCE), and to estimate the marginal willingness to pay (MWTP) associated with each attribute.

**Design:**

Six key attributes influencing responsiveness in oral health services were identified and refined through a literature review, pilot testing and expert consultation: clinic cleanliness, dentist specialisation, dentist attitude, clarity of explanation, treatment initiation time and contribution fee. To enable independent (orthogonal) estimation of attribute effects, minimise confounding due to interactions and ensure balanced representation of factor levels, an orthogonal fractional factorial design was employed to construct the DCE questionnaire. Data were collected using paper-based questionnaires, and MWTP estimates were derived from the resulting regression coefficients.

**Setting:**

The DCE was conducted at Hacettepe University’s Beytepe Campus in Ankara, Türkiye, between April and May 2024.

**Participants:**

375 administrative staff actively work at Hacettepe University’s Beytepe Campus.

**Main outcome measures:**

A conditional logit model was used to estimate preferences and MWTP for different attribute levels.

**Results:**

The attribute with the highest MWTP was the provision of specialised oral health services ($12.26), followed by clinic cleanliness, a concerned dentist, timely treatment initiation, clear explanations provided by the dentist and contribution fee. Upon incorporating interaction terms—namely age, gender, equivalised disposable income, preferred clinic type and alcohol use—significant variations in preferences were observed across subgroups.

**Conclusions:**

Policy responsiveness in oral healthcare requires identifying prioritised non-clinical service attributes. Policymakers must integrate this evidence into resource allocation and service planning. DCEs across diverse populations are essential for adapting service delivery to evolving public priorities and optimising care quality.

**STRENGTHS AND LIMITATIONS OF THIS STUDY**

- While focus group interviews are widely recommended for attribute development in discrete choice experiments (DCEs), this study relied on structured literature review and pilot testing, which may have limited initial exploration of context-specific concepts.
- A limited number of attributes was selected to minimise respondent burden and maintain cognitive feasibility; however, other related dimensions of oral health services may have been excluded.
- Due to the single-centre design and non-randomised sampling, generalisability is limited; however, the sample’s equivalised disposable income aligns with Türkiye’s, supporting economic representativeness.
- Preferences were quantified using marginal willingness to pay estimates, which enhance interpretability and support cross-study and policy-relevant comparisons.

## Introduction

 Oral health is a critical dimension of overall human well-being. Oral pathologies not only compromise local oral function but are also aetiologically linked to systemic conditions, including cardiovascular and respiratory diseases, diabetes and mental health disorders.^1^ Recognised as a major global public health challenge, oral diseases affect nearly half of the world’s population (approximately 3.5 billion individuals), despite being largely preventable. Furthermore, oral health status significantly influences multifaceted aspects of life, including psychological well-being, self-esteem, quality of life, productivity and social engagement.^2^

The primary objective of a health system is to enhance health outcomes. Nevertheless, the unpredictable and substantial costs associated with healthcare necessitate the implementation of risk-sharing and financial protection mechanisms to ensure equitable contributions to healthcare financing. Moreover, responsiveness to expectations beyond the clinical scope reinforces respect for individuals’ dignity, autonomy and confidentiality.^3^ Responsiveness is defined as ‘*…the outcome that can be achieved when institutions and institutional relationships are structured to recognize and appropriately respond to the universally legitimate expectations of individuals’*. The WHO posits that responsiveness should be examined from two perspectives: as a strategy to attract a larger consumer base, given that health service users are often conceptualised as customers, and as a means to safeguard patients’ rights to adequate and timely care.^4^ Health system responsiveness is shaped by both individual expectations and systemic factors, and a clear articulation of these expectations enables health system actors to respond effectively.^5^ Considering these factors, it is imperative to critically analyse public preferences for oral health services, with discrete choice experiments (DCEs) representing a promising methodological approach.

The DCE is a quantitative stated-preference method used to evaluate the relative importance of attributes defining products, services or policies, thereby elucidating the decision-making processes of diverse agents (eg, managers, consumers and voters).^6^ Within the DCE framework, respondents select their preferred alternative from sets of hypothetical options, each characterised by distinct attribute levels, including health outcomes and/or costs. This method enables the assignment of utility values to specific interventions or attributes and facilitates the calculation of their monetary value. Incorporating a monetary attribute (eg, a fee) into the DCE design permits the estimation of respondents’ marginal willingness to pay (MWTP) for incremental changes in service attributes.^7^ This derived MWTP metric serves as a critical input for cost-benefit analysis (CBA).^8^ CBA provides a common monetary valuation framework, allowing comparisons across disparate sectors (eg, transportation infrastructure, healthcare and public safety) by quantifying the monetary value that individuals place on different attributes. DCE-based CBA directly reveals individuals’ preferences for services, generating essential evidence for decision-makers to optimise resource allocation.

Although few studies have employed the DCE method to assess public preferences for oral health services as a responsiveness factor, it is essential for decision-makers to conduct these evaluations across different time points and contexts. Addressing this gap in the literature, this study aimed to evaluate individual preferences that influence responsiveness in oral health services using the DCE method.

## Methods and analysis

This study adhered to the guidelines and regulations of the International Society of Pharmacoeconomics and Outcomes Research, which establishes best practices for standardising conjoint analysis in healthcare research.^9^ Although the Discrete Choice Experiment Reporting Checklist had not been published at the time of the study, its recommendations were followed on publication (online supplemental file 1).^10^

### Selection of attributes and levels

In a DCE, participants evaluate hypothetical choice sets, each comprising multiple attributes and levels. These sets present two or more alternatives, which are assessed based on their attribute levels, allowing participants to select their preferred option. This method is widely used to analyse decision-making and preference structures across various fields.^11^

The initial list of 12 attributes was developed through a comprehensive literature review of previous DCE studies in oral health. These attributes included factors such as waiting time,^12^,^14^ dentist behaviour,^12 13 15 16^ clinic cleanliness,^12 17^ clarity of explanation,^12^ access to online consultation,^18^ distance to the clinic,^16 18^ immediacy of treatment,^18^ appointment convenience,^13^,^15^ treatment options,^15^ dentist specialisation^19^ and total treatment duration.^20^

To ensure contextual relevance and comprehensibility, a pilot study was conducted with 25 academic and administrative staff members outside the target sample. Participants were asked to evaluate 11 attributes and their levels. The process was jointly reviewed with a public health expert specialising in oral health to assess conceptual clarity and contextual appropriateness. Based on participant feedback and expert input, the five most consistently prioritised attributes were selected. A contribution fee attribute was later added to allow for MWTP estimation. Thus, a final set of six attributes was determined, in line with best practice recommendations for DCE design (typically 5–13 attributes) (table 1).^21^

**Table 1:** Attributes and attribute levels

Attributes	1st level	2nd level	3rd level
Cleanliness of the clinic	Not clean	Clean	–
The dentist’s specialisation in a particular field	Not available	Available	–
Attitude of the dentist	Not concerned	Concerned	
Clarity of the dentist’s explanation	Not clear	Clear	
Treatment initiation time	Not on time	On time	–
Contribution fee	–	25 Turkish liras	50 Turkish liras

In 2024, the Social Security Institution did not impose a contribution fee for primary care dental consultations. However, for secondary and tertiary healthcare services, a contribution fee ranging from 6 to 15 Turkish lira (TRY) was required. Anticipating future increases, the values 0, 25 and 50 were selected to facilitate calculation and enhance participants’ ability to distinguish between different fee levels. Following the completion of this study, in 2025, primary care services remained exempt from contribution fees, while contribution fees for secondary and tertiary healthcare services ranged from 20 to 50 TRY.^22^

### Experimental design and questionnaire

In the DCE, two unlabelled alternatives, ‘Clinic A’ and ‘Clinic B’, were presented alongside a ‘Neither’ option for respondents who preferred neither alternative. Selecting ‘Neither’ does not indicate a rejection of oral healthcare services but rather a preference outside the given choices. While the literature suggests that respondents may be more inclined to choose ‘Neither’ due to the DCE structure, forcing a selection between two alternatives could introduce bias. Thus, incorporating an ‘opt-out/status quo’ option is recommended to enhance response accuracy and minimise bias.^23 24^

A full factorial design includes all possible combinations of attribute levels, calculated by multiplying the number of levels across each attribute. In this study, five attributes had two levels each, and the contribution fee had three levels, resulting in 96 possible combinations. However, due to the impracticality of administering such a large number of choice sets, a fractional factorial design was employed. This approach strategically selects a subset of the full factorial design while retaining the ability to estimate key effects accurately. A total of 24 choice sets were generated using this fractional design.

Nevertheless, the number of choice sets each participant was required to complete remained relatively high. To address this, the 24 choice sets were divided into 2 blocks using the block method, resulting in a questionnaire containing 12 choice sets per participant. Due to the use of two blocks, two separate questionnaire forms—Form 1 and Form 2—were created, and each participant was assigned only one of them. This adjustment helped reduce cognitive burden, thereby improving response efficiency while preserving the integrity of the experimental design.^25^

Factorial designs enable the simultaneous investigation of multiple factors’ effects on a response variable; however, full factorial designs become increasingly impractical as the number of factors grows due to the exponential increase in experimental conditions. Fractional factorial designs address this limitation by selecting a strategically reduced subset of combinations. Among these, orthogonal fractional factorial designs are distinguished by their statistical efficiency: they allow for independent (orthogonal) estimation of factor effects, minimise confounding with interactions and ensure balanced representation of factor levels across experimental runs. Such designs are particularly valuable when resource constraints necessitate reduced run sizes without compromising the integrity of effect estimation.^26^

Mixed orthogonal designs are orthogonal arrays in which different factors have varying numbers of levels. In this study, five attributes had two levels each, while one attribute—the contribution fee—had three levels. Therefore, the design qualifies as a mixed orthogonal array. Accordingly, mixed orthogonal arrays were generated using the *support.CEs* package in R software. This package was employed to leverage its specialised functions for designing and analysing choice experiments, including design creation and randomisation procedures.^27^ The package implements randomisation via a mix-and-match method, an adaptation of the rotation technique. In this approach, one or more additional sets of N alternatives are derived from an orthogonal main-effect array and assigned to an urn. A single alternative was randomly selected from each urn to form a choice set. This selection process is iterated without replacement until all alternatives are assigned to exactly N choice sets. The resulting N choice sets collectively constitute the experimental design for the choice study.

This study employed the *support.CEs* package to generate an orthogonal fractional factorial design comprising 2 blocks of 12 choice sets. The design incorporated 6 attributes (each with 2–3 levels), 3 alternatives (‘Clinic A’, ‘Clinic B’, and ‘Neither’) and 24 choice sets (12 per block). Both Form 1 and Form 2 versions of the DCE questionnaire are provided in onlinesupplemental files 2  3.

### Study sample and survey administration

Multiple sample size calculation methodologies exist for DCEs. Orme’s formula, a widely cited approach, stipulates (n×t× a)/c≥500, where^28^:

- n=number of respondents,
- t=number of choice sets per respondent,
- a=number of alternatives per choice set (excluding the opt-out option),
- c=the largest number of levels for any single attribute.
  Applying this framework to the present study’s design (t=12, a=2, c=3) yields:

(n×12×2)/3≥500 ⟹ n≥62.5

Thus, the theoretical minimum sample size was 63 respondents. However, this threshold is inadequate for subgroup analyses and robust statistical inference. Empirical DCE studies typically recruit 100–300 participants to ensure analytical stability and reliability.^29^ Consequently, this study targeted a sample size exceeding the minimum threshold.

This study employed a convenience sampling approach to enrol all administrative staff at Hacettepe University’s Beytepe Campus in Ankara, Türkiye. Questionnaires were prepared in two distinct versions (Form 1 and 2) corresponding to the orthogonal design blocks, with each participant completing only one version. Data were collected using self-administered paper-based questionnaires between April and May 2024. The exclusion criteria targeted unreliable or inconsistent response patterns, while partial non-response was permitted. From a total administrative staff population of 1382 individuals, 375 eligible participants completed the survey, yielding a response rate of 27.1% and a final analytical sample of 375 respondents. All collected responses were retained for analysis based on the completed items.

### Statistical analysis

Data analysis was conducted using R Statistical Software V.4.4.1.^30^ Equivalised disposable income (EDI) was calculated according to the OECD (Organization for Economic Co-operation and Development) modified scale, applying coefficients of 1.0 to the household head, 0.5 to additional members aged ≥14 years and 0.3 to those <14 years.^31^ The EDI was derived by dividing the total monthly household income by the sum of the equivalence coefficients. While survey instruments displayed fees in TRY, all monetary values in the analysis were standardised to United States dollar (US$). For TRY-US$ conversion, the exchange rate announced by the Central Bank of the Republic of Türkiye during the study period was used as the reference (1 US$=32.50 TRY).^32^

In the DCEs, attributes such as ‘Cleanliness of the clinic’, ‘The dentist’s specialisation in a particular field’, ‘Attitude of the dentist’, ‘Clarity of the dentist’s explanation’ and ‘Treatment initiation time’ were categorical and thus dummy-coded. The ‘contribution fee’ was the sole continuous attribute, defined as an additional charge that a patient pays for a health service beyond the amount covered by the insurance.

To analyse respondents’ preferences for oral health services, conditional logistic regression was conducted using the *survival* package.^33^ Model goodness-of-fit parameters and MWTP were estimated. In this model, the MWTP is computed as the negative ratio of the estimated non-monetary coefficient to the monetary coefficient. The relative importance of each attribute level was calculated based on the percentage distribution of the total MWTP ranges. Significance level of α=0.05 was adopted for this study.

### Ethics and dissemination

In accordance with the principles of the Declaration of Helsinki, this study was conducted with prior ethical approval from the Social Sciences and Humanities Research Ethics Board of Hacettepe University (approval date: 19 March 2024, approval number: 2024/06).

### Patient and public involvement

Individuals participated in this study as research participants; however, they did not engage in its conceptualisation, design, recruitment or interpretation.

## Results

Table 2 presents a summary of the descriptive statistics for the 375 respondents. The percentages were computed based on valid responses. Regarding sociodemographic characteristics, 53.5% of respondents were female, 48.2% were between 39 and 51 years of age, 70.6% were married, 43.9% held a bachelor’s degree, 89.8% lived with family members, 61.6% refrained from alcohol consumption and 55.0% were either regular or former tobacco users. The average age was 44.07±9.52 years, with the youngest respondent being 19 years old and the oldest, 64 years old.

**Table 2:** Sociodemographic and oral health characteristics of respondents

Sociodemographic characteristics	n (%)	Oral health-related characteristics	n (%)
Gender		Frequency of tooth brushing	
Female	200 (53.5%)	≥Two times per day	200 (53.8%)
Male	174 (46.5%)	Less frequent	172 (46.2%)
Age		Frequency of changing the toothbrush	
≤38	97 (26.4%)	≥Once every 3 months	203 (59.0%)
39–51	177 (48.2%)	Less frequent	141 (41.0%)
≥52	93 (25.4%)	Frequency of using mouthwash	
Marital status		≥Once every 3 days	80 (21.6%)
Married	262 (70.6%)	Less frequent	290 (78.4%)
Single	92 (24.8%)	Use of dental floss/interfacial brush	
Other	17 (4.6%)	None	197 (53.2%)
Education		Only dental floss	95 (25.7%)
Elementary school	5 (1.3%)	Only interfacial brush	47 (12.7%)
Secondary school	9 (2.4%)	Both	31 (8.4%)
High school	67 (17.9%)	Mostly preferred clinic	
Associate degree	66 (17.7%)	Public dental clinic	184 (51.1%)
Bachelor’s degree	164 (43.9%)	Private dental clinic	176 (48.9%)
Master’s/doctoral degree	63 (16.8%)	Last time to visit the dentist	
Household composition		Within the last year	212 (57.0%)
With family members	335 (89.8%)	Other	160 (43.0%)
Alone	31 (8.3%)	Assessing own oral health	
With friends	7 (1.9%)	Very good/Good	100 (26.9%)
Alcohol consumption		Moderate	206 (55.3%)
Not drinkers	228 (61.6%)	Very bad/Bad	66 (17.8%)
Drinkers	142 (38.4%)	Diagnosed chronic disease	
Use of tobacco products		No	235 (63.7%)
Regular/former users	204 (55.0%)	Yes	134 (36.3%)
Do not use it or have only tried it	167 (45.0%)		

Regarding oral health characteristics, 53.8% of respondents reported brushing their teeth at least two times per day, while 59.0% replaced their toothbrushes at intervals not exceeding 3 months. Additionally, 78.4% used mouthwash less frequently than once every 3 days, and 53.2% did not use dental floss or an interdental brush. Moreover, 51.1% of the participants expressed a preference for public dental clinics, 57.0% had visited a dentist within the past year, 55.3% rated their oral health as moderate and 63.7% had not been diagnosed with a chronic disease.

The mean EDI was 1026.17±461.01 US$, with a median of 946.74 US$ and first and third quartiles of 700.37 and 1230.77 US$, respectively. Among regular and former tobacco users, the average number of cigarette pack-years was 15.22 (SD=16.06), with a median of 12.50 and first and third quartiles at 5.00 and 20.00, respectively.

The attribute with the highest MWTP was the availability of specialised services, estimated at $12.26, accounting for 28.48% of the total relative importance based on MWTP ranges (table 3 and online supplemental file 4). The remaining attributes, ranked in descending order of MWTP, were: a clean clinic environment ($11.39; 26.71%), a concerned dentist ($7.64; 18.36%), timely initiation of treatment ($6.20; 14.65%) and the dentist’s clear explanation of procedures ($5.00; 11.80%).

**Table 3:** Conditional logistic regression analysis of attribute levels and marginal willingness to pay (excluding interaction terms)

Attributes	Levels	β coefficients (SE)	MWTP (95% CI) (in US$)
The clinic’s cleanliness	Not clean	Ref.	
	Clean	1.06*** (0.05)	11.39 (5.63–54.66)
Provision of specialised services	Not available	Ref.	
	Available	1.14*** (0.06)	12.26 (6.14–58.42)
Attitude of the dentist	Not concerned	Ref.	
	Concerned	0.71*** (0.05)	7.64 (3.64–37.34)
Clarity of the dentist’s explanation	Not clear	Ref.	
	Clear	0.46*** (0.05)	5.00 (2.31–23.97)
Treatment initiation time	Not on time	Ref.	
	On time	0.58*** (0.05)	6.20 (2.93–29.83)
Contribution fee		−0.09* (0.04)	

*p<0.05, **p<0.01, ***p<0.001.

ASC (SE)=−2.51 (0.09), (p<0.001).

Log likelihood at start=−4825.11.

Log likelihood at convergence=−4128.44.

Log likelihood ratio χ2=1393 (df: 7), (p<0.001).

McFadden’s R2=0.144.

McFadden’s adjusted R2=0.143.

Akaike Information Criterion=8270.88.

Bayesian Information Criterion=8315.59.

Number of events=4392.

ASC, alternative specific constant; MWTP, marginal willingness to pay; Ref, reference; US$, United States dollar.

To achieve optimal model fit and uncover the demographic and behavioural factors influencing individual preferences—as well as to generate deeper policy insights—interaction terms were incorporated into the model. These included age, gender, EDI, preferred clinic type and alcohol use (adjusted R²=0.1725, Akaike Information Criterion=6327.7, Bayesian Information Criterion=6555.4; see online supplemental file 5). The inclusion of these terms increased the model’s explanatory power, as measured by McFadden’s adjusted R^2^, from 14.30% to 17.25%.

The analysis was confined to 290 respondents who provided complete data (table 4). Results indicated that individuals under 44 years were willing to pay $0.42 more than older adults for a concerned dentist (online supplemental file 6). Similarly, those preferring public clinics were willing to pay $0.42 more for a concerned dentist and $0.49 more for clearer explanations compared with private clinic users. Additionally, non-alcohol consumers were willing to pay $0.56 more for specialised services than alcohol consumers. These findings indicate that willingness to pay for specific attribute levels varies across sociodemographic and behavioural profiles.

**Table 4:** Coefficient estimates and goodness-of-fit statistics from conditional logistic regression analysis with interaction terms

Attributes	β coefficients (SE)					
	At start	**≤44 years**	Male	EDI ≤946.74 US$	Public centre	Drinking alcohol
Clean clinic	1.18*** (0.15)	0.13 (0.12)	−0.05 (0.11)	0.14 (0.12)	0.05 (0.12)	−0.21 (0.12)
Specialised services are available	1.27*** (0.15)	0.21 (0.12)	0.05 (0.12)	−0.01 (0.12)	0.11 (0.12)	−0.31** (0.12)
Concerned dentist	0.30* (0.14)	0.23* (0.11)	0.14 (0.11)	0.15 (0.11)	0.23* (0.11)	0.10 (0.11)
The dentist’s explanation is clear	0.38** (0.14)	−0.19 (0.11)	0.15 (0.11)	0.21 (0.12)	0.27* (0.11)	−0.14 (0.11)
Treatment initiated on time	0.68*** (0.14)	−0.13 (0.11)	0.03 (0.11)	−0.07 (0.11)	−0.13 (0.11)	0.10 (0.11)
Contribution fee	−0.55*** (0.12)	−0.07 (0.09)	0.16 (0.09)	0.29** (0.10)	0.14 (0.09)	0.13 (0.09)

Bold entries indicate statistically significant coefficients.

*p<0.05, **p<0.01, ***p<0.001.

ASC (SE)=−2.71 (0.10), (p<0.001).

Log likelihood at start=−3823.2.

Log likelihood at convergence=−3126.8.

Log likelihood ratio χ2=1393 (df:37), (p<0.001).

McFadden’s R2=0.182.

McFadden’s adjusted R2=0.172.

Akaike Information Criterion=6327.7.

Bayesian Information Criterion=6555.4.

Number of events=3480.

ASC, alternative specific constant; EDI, equivalised disposable income; US$, United States dollar.

Changes in MWTP values following the inclusion of interaction terms are attributable to shifts in the underlying utility coefficients. These shifts reflect conditional effects that vary across respondent characteristics, thereby enhancing the model’s explanatory power and sensitivity to subgroup differences.

**Figure 1:**
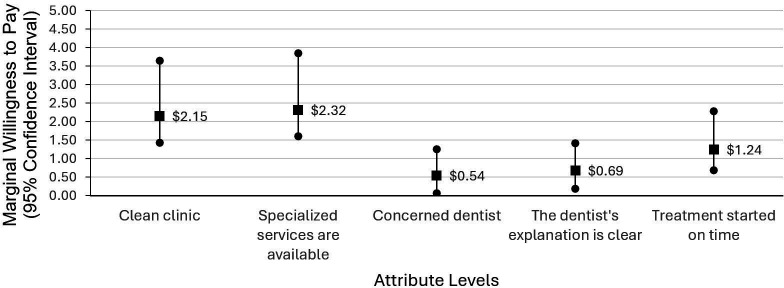
Marginal willingness to pay (95% CIs) accounting for interaction effects of age, gender, equivalised disposable income, preferred centre and alcohol use.

Respondents were willing to pay an additional $2.32 (95% CI $1.61 to $3.85) for specialised services, $2.15 (95% CI $1.43 to $3.65) for a clean clinic, $1.24 (95% CI $0.68 to $2.28) for timely treatment, $0.69 (95% CI $0.18 to $1.42) for clear explanations by the dentist and $0.54 (95% CI $0.06 to $1.26) for a concerned dentist (figure 1). When interaction terms were included, specialised services and clean clinic continued to elicit the highest MWTP, followed by timely treatment. Notably, clear explanation by the dentist exceeded concern shown by the dentist, reflecting a shift in the relative valuation of interpersonal service features.

## Discussion

Understanding how society evaluates health interventions is essential for both clinical decision-making and strategic planning. In this study, oral health behaviours—such as tooth brushing, toothbrush replacement, mouthwash use and dental visits—were found to occur at notably low frequencies. Additionally, the high prevalence of tobacco use poses a significant risk to oral health. Enhancing public engagement and improving the effectiveness of oral health services requires a clear understanding of societal preferences. While the cost is widely recognised as a significant determinant of decision-making, individual preferences for other characteristics may also influence choices.^34^

This study employed a DCE to examine individual preferences influencing responsiveness to oral health services. The highest MWTP was observed for specialised oral health services, followed by clinic cleanliness, timely treatment initiation, clear communication of diagnoses and treatment plans, dentist attitude and contribution fee. These findings suggest that service planning should reflect this hierarchy of preferences to better align with user expectations. The elevated MWTP values for specialised services and cleanliness may indicate unmet needs in these areas, underscoring the importance of prioritising them in policy design. To address potential gaps in service delivery, strengthening monitoring and quality control mechanisms is recommended. Systematic evaluations can help identify deficiencies and guide evidence-based improvements.

Analysis of the interaction terms—including age, gender, EDI, clinic type preference and alcohol use—revealed notable variations in MWTP across sociodemographic subgroups for specific service attributes. These findings suggest divergent valuations of oral health service features among population segments.

For instance, younger individuals placed greater emphasis on dentist concern, highlighting the need for decision-makers and practitioners to prioritise this aspect when designing services for youth. Similarly, public clinic users valued dentist attitude and explanation clarity more strongly, which may reflect deficiencies in these dimensions within public facilities compared with private ones. Addressing these gaps may require further investigation into dentist-patient communication, provision of in-service training for practitioners and adjustments to consultation durations. Accounting for such heterogeneity is essential for healthcare planners and policymakers to ensure that service design and delivery are responsive to diverse population needs.

International studies corroborate this finding. A Brazilian study (2013–2014) associated patients’ positive structural assessments of primary oral care centres with increased satisfaction regarding their dentists.^35^ Similarly, research at a Croatian dental school clinic (2016–2017) identified patient priorities as comprehensive treatment explanations, cordial staff conduct, reduced waiting periods and affordability.^36^ A 2018 UK investigation into private adult orthodontics found that practitioner qualifications and expertise outweighed appliance type, commanding the highest willingness to pay.^37^ Furthermore, a 2019 Romanian study of adult patients found that dentist expertise was the primary criterion in selecting oral healthcare facilities. Sterilisation standards, strict infection control protocols and staff courtesy were also prioritised over location and treatment costs.^38^ These international findings align with our results and suggest that such attributes remain important across different regions and time periods. It is therefore essential for policymakers to consider these factors when planning oral health services. Doing so may enhance the effectiveness and responsiveness of service delivery.

Our results correspond with those of national studies conducted across distinct time periods. A 2020 investigation of patients at a Turkish dental hospital faculty identified treatment quality, practitioner and assistant personal attributes, service accessibility, facility conditions and cost as the multidimensional determinants of preference.^16^ This aligns with earlier Turkish research (2004), which established privacy and practitioner demeanour as factors of paramount importance for satisfaction.^12^

A comparative analysis reveals a notable divergence. In a DCE conducted at a Croatian dental school (2016–2017), nearly half of the participants disregarded cost as an attribute—unlike our sample, which clearly valued contribution fees.^39^ This variation may be associated with the extent to which oral health services are covered by insurance or with differences in EDI, suggesting that contextual and methodological factors can influence stated preferences.

### Strengths and limitations

Pilot testing was employed in this study, although focus group interviews are widely recommended for attribute development in DCEs. While pilot testing ensured cognitive clarity and contextual appropriateness, the absence of qualitative exploration may have constrained the initial conceptual scope. Certain dimensions of responsiveness—particularly those shaped by patient narratives or culturally embedded expectations (eg, waiting time)—may not have been sufficiently captured. Incorporating formative qualitative methods in future research could enhance construct validity and support more comprehensive attribute identification.

A limited number of attributes was selected to reduce cognitive burden and maintain response quality, particularly given the relatively high number of choice tasks per participant. However, this restriction may have excluded other relevant aspects of oral health services that may have influenced individual preferences.

This study contributes to the limited body of national research by applying a DCE to assess preferences for responsiveness-related attributes in oral health services. In the absence of comparable domestic DCE studies, benchmarking opportunities were limited. Nonetheless, the findings offer a valuable foundation for future methodological applications in Türkiye.

Applying DCE methodology to oral health responsiveness—an underexplored dimension in public health—may encourage its application for analogous priority topics in health services research and policy. Using open-source computational tools (eg, R statistical environment) with explicit documentation of analytical packages further promotes methodological transparency and reproducibility within the scientific community.

In line with this approach, this study used a conditional logit model, which is well-suited for analysing discrete choice data and yielded valuable insights into population-level preferences. Nonetheless, to more effectively capture preference heterogeneity, advanced modelling approaches such as mixed multinomial logit and latent class analysis may be considered.

The study sample exhibited a balanced gender distribution, with nearly equivalent proportions of male and female participants. However, this apparent homogeneity contrasts with the observed socioeconomic heterogeneity, evidenced by the disproportionate representation of married individuals, bachelor’s degree holders and those cohabiting with family members. This demographic profile may reflect recruitment dynamics, whereby such individuals are more likely to be available during standard working hours, more willing to participate in research activities, and potentially more familiar with structured survey formats such as DCEs.

Since our study was conducted in a single centre and a non-randomised convenience sample, the results cannot be generalised to the entire population. However, according to the income distribution statistics, the annual EDI in 2024 is 374 899.00 TRY, which corresponds to 31 241.58 TRY per month ($961.28), whereas the EDI in our sample is $946.74.^40^ This close alignment suggests that, despite limited geographic coverage, the sample is economically comparable to the national population, supporting contextual relevance. Moreover, the use of convenience sampling reflects the prevailing recruitment strategy in oral health choice experiments, as documented in recent systematic reviews.^41^ In addition, inherent limitations of DCE methodology—such as cognitive fatigue, unfamiliarity with the format and the time required to complete choice tasks—may have influenced response behaviour, as commonly observed in similar studies.

Preferences were quantified using MWTP estimates, which enhance interpretability by translating abstract utility values into monetary terms. This approach facilitates clearer communication of trade-offs and supports meaningful comparisons across studies, populations and healthcare settings. Moreover, expressing preferences in monetary units may inform resource allocation decisions and contribute to policy-relevant insights in oral health service planning.

## Conclusion

Policy responsiveness in oral healthcare requires identifying prioritised non-clinical service attributes. Our findings establish a preference hierarchy led by specialised services, followed by clinic cleanliness, treatment timeliness, the dentist’s explanations, the dentist’s attitude and contribution fee. Sociodemographic factors shape these preferences. Policymakers must integrate this evidence into resource allocation and service planning. DCEs across diverse populations are essential for adapting service delivery to evolving public priorities and optimising care quality.

## Supplementary material

10.1136/bmjopen-2025-106411 online supplemental file 1

10.1136/bmjopen-2025-106411 online supplemental file 2

10.1136/bmjopen-2025-106411 online supplemental file 3

10.1136/bmjopen-2025-106411 online supplemental file 4

10.1136/bmjopen-2025-106411 online supplemental file 5

10.1136/bmjopen-2025-106411 online supplemental file 6

## Data Availability

The data that support the findings of this study are not openly available due to reasons of sensitivity and the deidentified participant data are available from the corresponding author upon reasonable request.
