# Supplementary material for: What influences individual preferences for responsiveness in oral health services? A discrete choice experiment in Türkiye
Source: BMJ Open. 2025 Nov 21;15(11):e106411. doi: 10.1136/bmjopen-2025-106411 (PMC12658521; doi:10.1136/bmjopen-2025-106411)
Supplement: online supplemental file 1 [file bmjopen-15-11-s001.docx]

**Table S1** Checklist for reporting discrete choice experiments in health (The DIRECT checklist)

| **Section Item** |  | **Page and paragraph** |
| --- | --- | --- |
| Purpose and rationale |  |  |
| 1 | Describe the real-world context and decision-maker that the hypothetical choice context seeks to replicate or inform | Introduction-Paragraph 1, 2 |
| 2 | Provide a rationale for using a DCE to answer the research question | Introduction-Paragraph 2, 3 |
| Attributes and levels |  |  |
| 3 | Describe how attributes and levels were derived (e.g. literature review, interviews, focus groups, expert input) | Methods and Analysis, Selection of attributes and levels-Paragraph 1-5 |
| 4 | Provide the final list of attributes and levels | Table 1 |
| Experimental design |  |  |
| 5 | Report the number of alternatives per choice set and whether they were labelled or unlabelled | Methods and Analysis-Experimental design and questionnaire-Paragraph 1 |
| 6 | Describe response options (e.g. forced choice, opt-out, status quo) | Methods and Analysis-Experimental design and questionnaire-Paragraph 1 |
| 7 | Describe the type of experimental design (e.g. orthogonal, D-efficient, Bayesian efficient, partial profile) | Methods and Analysis-Experimental design and questionnaire-Paragraph 4-5 |
| 8 | Describe which effects are identified in the design (e.g. main effects, higher order interactions, functional form) | Methods and Analysis-Experimental design and questionnaire-Paragraph 4-5 |
| 9 | Describe the number of choice sets, blocks and choice sets per block | Methods and Analysis-Experimental design and questionnaire-Paragraph 6 |
| 10 | Indicate how the experimental design was obtained (software, catalogue, other) | Methods and Analysis-Experimental design and questionnaire-Paragraph 5-6 |
| Survey design |  |  |
| 11 | Provide a sample choice set and the instructions and background information given to respondents (e.g. providing the survey as an appendix) | Supplementary File 2-3. |
| 12 | Report any randomization (e.g. choice set order, attribute order, alternative order, framing effects) | Methods and Analysis-Experimental design and questionnaire-Paragraph 5 |
| 13 | Describe what was checked in piloting (e.g. understanding, respondent burden, timing, wording) | Methods and Analysis-Selection of attributes and levels section-Paragraph 3-4 |
| 14 | Report whether information from the pilot was used to update the experimental design (e.g. priors, functional form of attributes) or survey design | Methods and Analysis-Selection of attributes and levels section-Paragraph 3-4 |
| Sample and data collection |  |  |
| 15 | Report respondent inclusion/exclusion criteria | Methods and Analysis-Study sample and survey administration-Paragraph 4 |
| 16 | Describe how data were collected (e.g. mail, personal interview, web survey) | Methods and Analysis-Study sample and survey administration-Paragraph 4 |
| 17 | Report the response rate or cooperation rate, if possible | Methods and Analysis-Study sample and survey administration-Paragraph 4 |
| 18 | Report the final sample size and how the sample size was determined | Methods and Analysis-Study sample and survey administration-Paragraph 1-4 |
| 19 | Describe respondent characteristics and representativeness of target population, if known | Table 2, Results-Paragraph 1-3 |
| Econometric analysis |  |  |
| 20 | Indicate coding of data (e.g. effects, dummy, continuous) including definitions | Methods and Analysis-Statistical analysis-Paragraph 2 |
| 21 | Report whether any respondents were removed and why (e.g. suspected fraudulent responses, rationality tests) | Methods and Analysis-Study sample and survey administration-Paragraph 4 |
| 22 | Provide the rationale for model choice (e.g. conditional logit, mixed logit, latent class) and assumptions (e.g. error variance) | Methods and Analysis-Statistical analysis-Paragraph 3 |
| 23 | Report model specification | Table 3 and 4 |
| Reporting of results |  |  |
| 24 | Report the model performance, goodness of fit (if comparing models) | Table 3 and 4 |
| 25 | Describe methods used for analysis of model results (e.g. calculation of marginal rate of substitution, attribute relative importance, welfare gain) | Methods and Analysis-Statistical analysis-Paragraph 3, Results-Paragraph 4-8 |
| 26 | Report measures of precision for the output(s) of interest (e.g. confidence intervals) and how these were derived | Table 3 and 4 |

Source: Ride J, Goranitis I, Meng Y, LaBond C, Lancsar E. A Reporting Checklist for Discrete Choice Experiments in Health: The DIRECT Checklist. PharmacoEconomics. 2024;42:1161–75.
