# Supplementary material for: What influences individual preferences for responsiveness in oral health services? A discrete choice experiment in Türkiye
Source: BMJ Open. 2025 Nov 21;15(11):e106411. doi: 10.1136/bmjopen-2025-106411 (PMC12658521; doi:10.1136/bmjopen-2025-106411)
Supplement: online supplemental file 2 [file bmjopen-15-11-s002.pdf]

1) Which clinic do you prefer?

- ☐ Clinic A
- ☐ Clinic B
- ☐ Neither

	A	B
**Cleanliness of the clinic**	Not clean	Clean
**The dentist's specialization in a particular field**	Not available	Available
**Attitude of the dentist**	Not concerned	Concerned
**Clarity of the dentist's explanation**	Clear	Clear
**Treatment initiation time**	Not on time	On time
**Contribution fee**	50 TRY ₺ ₺	25 TRY ₺

---

2) Which clinic do you prefer?

- ☐ Clinic A
- ☐ Clinic B
- ☐ Neither

	A	B
**Cleanliness of the clinic**	Not clean	Clean
**The dentist's specialization in a particular field**	Not available	Available
**Attitude of the dentist**	Concerned	Not concerned
**Clarity of the dentist's explanation**	Not clear	Clear
**Treatment initiation time**	Not on time	On time
**Contribution fee**	25 TRY ₺	50 TRY ₺ ₺

DCE Questionnaire Form 1 (Block 1)

3) Which clinic do you prefer?

☐ Clinic A
☐ Clinic B
☐ Neither

	A	B
**Cleanliness of the clinic**	Not clean	Not clean
**The dentist's specialization in a particular field**	Available	Available
**Attitude of the dentist**	Not concerned	Concerned
**Clarity of the dentist's explanation**	Clear	Not clear
**Treatment initiation time**	On time	On time
**Contribution fee**	-	50 TRY ₺ ₺

---

4) Which clinic do you prefer?

☐ Clinic A
☐ Clinic B
☐ Neither

	A	B
**Cleanliness of the clinic**	Not clean	Not clean
**The dentist's specialization in a particular field**	Not available	Available
**Attitude of the dentist**	Concerned	Not concerned
**Clarity of the dentist's explanation**	Not clear	Not clear
**Treatment initiation time**	On time	Not on time
**Contribution fee**	25 TRY ₺	25 TRY ₺

5) Which clinic do you prefer?

- [ ] Clinic A
- [ ] Clinic B
- [ ] Neither

	A	B
**Cleanliness of the clinic**	Clean	Not clean
**The dentist's specialization in a particular field**	Available	Available
**Attitude of the dentist**	Concerned	Concerned
**Clarity of the dentist's explanation**	Not clear	Clear
**Treatment initiation time**	On time	Not on time
**Contribution fee**	50 TRY ₺ ₺	50 TRY ₺ ₺

---

6) Which clinic do you prefer?

- [ ] Clinic A
- [ ] Clinic B
- [ ] Neither

	A	B
**Cleanliness of the clinic**	Not clean	Not clean
**The dentist's specialization in a particular field**	Not available	Not available
**Attitude of the dentist**	Concerned	Not concerned
**Clarity of the dentist's explanation**	Clear	Clear
**Treatment initiation time**	On time	Not on time
**Contribution fee**	-	-

DCE Questionnaire Form 1 (Block 1)

7) Which clinic do you prefer?

- [ ] Clinic A
- [ ] Clinic B
- [ ] Neither

	A	B
**Cleanliness of the clinic**	Clean	Not clean
**The dentist's specialization in a particular field**	Not available	Not available
**Attitude of the dentist**	Not concerned	Concerned
**Clarity of the dentist's explanation**	Clear	Not clear
**Treatment initiation time**	Not on time	On time
**Contribution fee**	25 TRY ₺	50 TRY ₺ ₺

---

8) Which clinic do you prefer?

- [ ] Clinic A
- [ ] Clinic B
- [ ] Neither

	A	B
**Cleanliness of the clinic**	Clean	Clean
**The dentist's specialization in a particular field**	Not available	Available
**Attitude of the dentist**	Not concerned	Not concerned
**Clarity of the dentist's explanation**	Not clear	Not clear
**Treatment initiation time**	On time	Not on time
**Contribution fee**	25 TRY ₺	25 TRY ₺

DCE Questionnaire Form 1 (Block 1)

9) Which clinic do you prefer?

- ☐ Clinic A
- ☐ Clinic B
- ☐ Neither

	A	B
Cleanliness of the clinic	Clean	Clean
The dentist's specialization in a particular field	Available	Available
Attitude of the dentist	Not concerned	Concerned
Clarity of the dentist's explanation	Clear	Not clear
Treatment initiation time	On time	Not on time
Contribution fee	25 TRY	-

---

10) Which clinic do you prefer?

- ☐ Clinic A
- ☐ Clinic B
- ☐ Neither

	A	B
Cleanliness of the clinic	Clean	Clean
The dentist's specialization in a particular field	Available	Not available
Attitude of the dentist	Not concerned	Concerned
Clarity of the dentist's explanation	Not clear	Clear
Treatment initiation time	Not on time	Not on time
Contribution fee	-	-

DCE Questionnaire Form 1 (Block 1)

11) Which clinic do you prefer?

- [ ] Clinic A
- [ ] Clinic B
- [ ] Neither

	A	B
Cleanliness of the clinic	Clean	Clean
The dentist's specialization in a particular field	Not available	Not available
Attitude of the dentist	Concerned	Not concerned
Clarity of the dentist's explanation	Clear	Clear
Treatment initiation time	On time	Not on time
Contribution fee	-	50 TRY ₺ ₺

---

12) Which clinic do you prefer?

- [ ] Clinic A
- [ ] Clinic B
- [ ] Neither

	A	B
Cleanliness of the clinic	Not clean	Clean
The dentist's specialization in a particular field	Available	Available
Attitude of the dentist	Concerned	Concerned
Clarity of the dentist's explanation	Clear	Not clear
Treatment initiation time	Not on time	On time
Contribution fee	25 TRY ₺	-

DCE Questionnaire Form 1 (Block 1)
