# Supplementary material for: What influences individual preferences for responsiveness in oral health services? A discrete choice experiment in Türkiye
Source: BMJ Open. 2025 Nov 21;15(11):e106411. doi: 10.1136/bmjopen-2025-106411 (PMC12658521; doi:10.1136/bmjopen-2025-106411)
Supplement: online supplemental file 4 [file bmjopen-15-11-s004.docx]

**Figure S1** Relative importance of attributes in DCE (based on MWTP range)
