# Supplementary material for: What influences individual preferences for responsiveness in oral health services? A discrete choice experiment in Türkiye
Source: BMJ Open. 2025 Nov 21;15(11):e106411. doi: 10.1136/bmjopen-2025-106411 (PMC12658521; doi:10.1136/bmjopen-2025-106411)
Supplement: online supplemental file 5 [file bmjopen-15-11-s005.docx]

**Table S2** Model goodness-of-fit according to selected variables

| **Variables** | **Adj. R^2^** | | **AIC** | **BIC** |
| --- | --- | --- | --- | --- |
| Age+Gender+Smoking | 0.1521440 | | 7936.082 | 8095.007 |
| Age+Gender+Education+Smoking | 0.1526943 | | 7930.931 | 8127.998 |
| Age+Gender | 0.1530677 | | 7994.429 | 8115.372 |
| Age+Gender+Education | 0.1538606 | | 7986.944 | 8146.080 |
| Age+Gender+Center | 0.1553056 | | 7683.771 | 7841.982 |
| Age+Gender+Center+Education | 0.1559589 | | 7677.828 | 7874.010 |
| Age+EDI | 0.1666334 | | 6613.910 | 6731.558 |
| Age+Gender+EDI+Smoking+Alcohol | 0.1681466 | | 6514.168 | 6742.777 |
| Age+Gender+EDI+Education | 0.1681823 | | 6601.617 | 6793.570 |
| Age+Gender+EDI | 0.1685310 | | 6598.850 | 6753.651 |
| Age+Gender+EDI+Smoking | 0.1685965 | | 6554.487 | 6746.233 |
| Age+Gender+EDI+Alcohol | 0.1695597 | | 6524.998 | 6716.640 |
| Age+Gender+EDI+Center+Smoking+Alcohol | 0.1710542 | | 6316.546 | 6581.053 |
| Age+Gender+EDI+Center+Smoking | 0.1711765 | | 6359.321 | 6587.175 |
| Age+Gender+EDI+Center | 0.1712169 | | 6402.716 | 6593.833 |
| **Age+Gender+EDI+Center+Alcohol** | **0.1724578** | | **6327.670** | **6555.398** |
| Adj: Adjusted | | AIC: Akaike Information Criterion | | |
| BIC: Bayesian Information Criterion | | EDI: Equivalized Disposable Income | | |
