# Supplementary material for: What influences individual preferences for responsiveness in oral health services? A discrete choice experiment in Türkiye
Source: BMJ Open. 2025 Nov 21;15(11):e106411. doi: 10.1136/bmjopen-2025-106411 (PMC12658521; doi:10.1136/bmjopen-2025-106411)
Supplement: online supplemental file 6 [file bmjopen-15-11-s006.docx]

| **Table S3** Marginal Willingness to Pay of Attribute Levels with Interaction Terms   \| Attribute / Interaction \| MWTP ($) \| Lower 2.5% ($) \| Upper 97.5% ($) \| \| --- \| --- \| --- \| --- \| \| clean* \| **2.1442** \| **1.4328** \| **3.7347** \| \| available* \| **2.3166** \| **1.6117** \| **3.8906** \| \| concerned* \| **0.5436** \| **0.0524** \| **1.2517** \| \| clear* \| **0.6830** \| **0.1972** \| **1.4445** \| \| on time* \| **1.2437** \| **0.6925** \| **2.3142** \| \| clean:age \| 0.2449 \| -0.1783 \| 0.8225 \| \| available:age \| 0.3911 \| -0.0240 \| 1.0812 \| \| concerned:age* \| **0.4228** \| **0.0351** \| **1.0120** \| \| clear:age \| -0.3470 \| -0.8748 \| 0.0542 \| \| on time:age \| -0.2287 \| -0.7063 \| 0.1637 \| \| clean:EDI \| 0.2583 \| -0.1681 \| 0.8502 \| \| available:EDI \| -0.0168 \| -0.4763 \| 0.4852 \| \| concerned:EDI \| 0.2707 \| -0.1400 \| 0.8122 \| \| available:EDI \| 0.3786 \| -0.0592 \| 0.9746 \| \| on time:EDI \| -0.1318 \| -0.5935 \| 0.3091 \| \| clean:gender \| -0.0913 \| -0.5492 \| 0.3503 \| \| available:gender \| 0.0968 \| -0.3352 \| 0.6247 \| \| concerned:gender \| 0.2519 \| -0.1398 \| 0.7714 \| \| clear:gender \| 0.2785 \| -0.1333 \| 0.8326 \| \| on time:gender \| 0.0568 \| -0.3624 \| 0.4986 \| \| clean:alcohol \| -0.3778 \| -0.9045 \| 0.0486 \| \| available:alcohol* \| **-0.5585** \| **-1.1399** \| **-0.1397** \| \| concerned:alcohol \| 0.1811 \| -0.2067 \| 0.6815 \| \| clear:alcohol \| -0.2471 \| -0.7431 \| 0.1818 \| \| on time:alcohol \| 0.1847 \| -0.2076 \| 0.6734 \| \| clean:center \| 0.0848 \| -0.3555 \| 0.5695 \| \| available:center \| 0.2028 \| -0.2307 \| 0.7410 \| \| concerned:center* \| **0.4202** \| **0.0276** \| **0.9907** \| \| clear:center* \| **0.4926** \| **0.0755** \| **1.1540** \| \| on time:center \| -0.2323 \| -0.7123 \| 0.1539 \| \| EDI: Equivalized Disposable Income \| \| \| \| |
| --- | --- | --- | --- | --- | --- | --- | --- | --- | --- | --- | --- | --- | --- | --- | --- | --- | --- | --- | --- | --- | --- | --- | --- | --- | --- | --- | --- | --- | --- | --- | --- | --- | --- | --- | --- | --- | --- | --- | --- | --- | --- | --- | --- | --- | --- | --- | --- | --- | --- | --- | --- | --- | --- | --- | --- | --- | --- | --- | --- | --- | --- | --- | --- | --- | --- | --- | --- | --- | --- | --- | --- | --- | --- | --- | --- | --- | --- | --- | --- | --- | --- | --- | --- | --- | --- | --- | --- | --- | --- | --- | --- | --- | --- | --- | --- | --- | --- | --- | --- | --- | --- | --- | --- | --- | --- | --- | --- | --- | --- | --- | --- | --- | --- | --- | --- | --- | --- | --- | --- | --- | --- | --- | --- | --- | --- | --- | --- | --- |
