# Supplementary material for: What influences individual preferences for responsiveness in oral health services? A discrete choice experiment in Türkiye
Source: BMJ Open. 2025 Nov 21;15(11):e106411. doi: 10.1136/bmjopen-2025-106411 (PMC12658521; doi:10.1136/bmjopen-2025-106411)
Supplement: Supplementary file 7 [file bmjopen-2025-106411.reviewer_comments.pdf]

# PEER REVIEW HISTORY

BMJ Open publishes all reviews undertaken for accepted manuscripts. Reviewers are asked to complete a checklist review form and are provided with free text boxes to elaborate on their assessment. These free text comments are reproduced below.

## ARTICLE DETAILS

### Title (Provisional)

What Influences Individual Preferences for Responsiveness in Oral Health Services? A Discrete Choice Experiment in Türkiye

### Authors

Özbek, Murat; Yardım, Mahmut Saadi

---

## VERSION 1 - REVIEW

---

| | |
|---|---|
| **Reviewer** | **1** |
| **Name** | **Senanayake, Sameera** |
| **Affiliation** | **Queensland University of Technology, Duke-NUS Medical School** |
| **Date** | **31-Aug-2025** |
| **COI** | **None** |

---

Overall Comment

This is a clear and well-conducted study that addresses an important and under-researched topic in Turkey. The manuscript is well structured and the analysis is appropriate. However, providing additional clarifications and methodological details would further strengthen the transparency and impact of the study.

Specific Comments

1. The manuscript notes that the initial list of 12 attributes was generated through a literature review. In many DCE studies, this step is often followed by qualitative work (e.g., focus groups or interviews) to check whether any context-specific attributes have been overlooked and to confirm relevance for the target population. It would be helpful for the authors to explain why this step was not undertaken.

2. In addition, while the manuscript states that the 12 attributes were reduced to 6 based on “expert opinion,” more detail would improve clarity. For example, who were the experts (clinicians, researchers, service users), and how was their input incorporated? Providing this information would make the process of attribute selection more transparent.

3. On page 6, line 22, the statement "The block method is used when a full factorial design is too large to be presented entirely to each respondent" is misleading. Blocking is typically applied when a fractional factorial design is still too large for each respondent to complete.

4. In the Methods section, please report: (i) The total size of the experimental design (i.e., number of choice sets generated for the fractional factorial design); (ii) How many blocks were used, and how many tasks were presented per respondent. These details are essential for reproducibility and interpretation.

5. Page 9, line 54: The sentence "The attribute with the highest willingness-to-pay … was the provision of specialized services (ß coefficient [SE] = 1.14 [0.06], p<0.001)" is inaccurate. The reported value (1.14) is a coefficient estimate, not a willingness-to-pay value.

6. Page 9, line 57: The phrase "Subsequent attributes, in descending order of importance …" should be clarified. Attribute importance should ideally be quantified using relative importance measures, calculated from the utility ranges across attribute levels. Reporting relative importance would provide a more robust comparison of attribute salience. Relative importance of attributes is estimated by taking the range of utility values for each attribute (the difference between its highest and lowest level coefficients) and dividing it by the sum of all attribute ranges. This gives a percentage value for each attribute, showing how much it contributed to the overall choice compared with the others.

7. The use of conditional logit models is appropriate and provides valuable insights. At the same time, future work could be strengthened by incorporating more advanced approaches, such as mixed multinomial logit (MIXL) and latent class models, to better capture preference heterogeneity. The apollo package in R is well suited for implementing these models and could offer the authors greater flexibility in future analyses.

---


| | |
|---|---|
| **Reviewer** | **2** |
| **Name** | **Shahzad, Muhammad** |
| **Affiliation** | **Khyber Medical University** |
| **Date** | **07-Sep-2025** |
| **COI** | **None** |


---

The manuscript report DCE to assess attributes preferences in oral healthcare in Türkiye. Overall, the study design, methodology and reporting are clear. The study has attempted to address a relatively unexplored area of public health, especially in context of oral healthcare which may have important implications for policy making. However, there are some minor issues that need to be addressed before the it is accepted for publication.

1. The abstract is not clear with too much methodological details. Please rewrite and simplify.

2. Some words and phrases are too technical for non-technical readers e.g. orthogonal fractional factorial design. While I understand that these terminologies represent underlying concepts, if possible, please simplify or explain further.

3. Results section focus too much on coefficients. A more narrative interpretation will make it clear for lay readers.

4. The discussion section should focus more on comparison with already available literature and public health policy implications of the research, rather than statistical details.

5. The overall language is good. However, some sentences are overly complex and could be simplified.

---

## VERSION 1 - AUTHOR RESPONSE

Replies to Reviewer 1's Comments:

- The manuscript notes that the initial list of 12 attributes was generated through a literature review. In many DCE studies, this step is often followed by qualitative work (e.g., focus groups or interviews) to check whether any context-specific attributes have been overlooked and to confirm relevance for the target population. It would be helpful for the authors to explain why this step was not undertaken.
    - The previous statement has been revised and presented in its updated form on page 4, line 50.
- In addition, while the manuscript states that the 12 attributes were reduced to 6 based on "expert opinion," more detail would improve clarity. For example, who were the experts (clinicians, researchers, service users), and how was their input incorporated? Providing this information would make the process of attribute selection more transparent.
    - The previous statement has been revised and presented in its updated form on page 4, line 50.
- On page 6, line 22, the statement "The block method is used when a full factorial design is too large to be presented entirely to each respondent" is misleading. Blocking is typically applied when a fractional factorial design is still too large for each respondent to complete.
    - The previous statement has been revised and presented in its updated form on page 6, line 8.
- In the Methods section, please report: (i) The total size of the experimental design (i.e., number of choice sets generated for the fractional factorial design); (ii) How many blocks were used, and how many tasks were presented per respondent. These details are essential for reproducibility and interpretation.

- To enhance methodological transparency and reproducibility, we have revised the relevant section of the manuscript to explicitly report the total number of choice sets generated through the fractional factorial design, the number of blocks used, and the number of tasks presented to each respondent. These details are now clearly stated on page 6, lines 8 and 55 of the revised manuscript.

- Page 9, line 54: The sentence "The attribute with the highest willingness-to-pay … was the provision of specialized services (ß coefficient [SE] = 1.14 [0.06], p<0.001)" is inaccurate. The reported value (1.14) is a coefficient estimate, not a willingness-to-pay value.
  - The statement has been revised, and it was decided to report marginal willingness to pay values instead of beta coefficients. This change is reflected on page 10, line 14 of the revised manuscript.
- Page 9, line 57: The phrase "Subsequent attributes, in descending order of importance …" should be clarified. Attribute importance should ideally be quantified using relative importance measures, calculated from the utility ranges across attribute levels. Reporting relative importance would provide a more robust comparison of attribute salience. Relative importance of attributes is estimated by taking the range of utility values for each attribute (the difference between its highest and lowest level coefficients) and dividing it by the sum of all attribute ranges. This gives a percentage value for each attribute, showing how much it contributed to the overall choice compared with the others.
  - To enhance transparency, we calculated relative importance scores using MWTP ranges across attribute levels, normalized to sum to 100%. This MWTP-based approach aligns with the interpretation used throughout the paper and is now reflected in the main results (page 10, line 14) and Supplementary File 4. As the cost attribute is continuous, its utility range is not directly comparable to that of categorical attributes using the conventional method, which further supports the use of MWTP-based importance measures.
- The use of conditional logit models is appropriate and provides valuable insights. At the same time, future work could be strengthened by incorporating more advanced approaches, such as mixed multinomial logit (MIXL) and latent class models, to better capture preference heterogeneity. The apollo package in R is well suited for implementing these models and could offer the authors greater flexibility in future analyses.

- We thank the reviewer for this insightful recommendation. We will keep it in mind for our future work. To reflect its relevance for future research, we have addressed it under the "Strengths and limitations" section (page 14, line 31).

Replies to Reviewer 2's Comments:

- The abstract is not clear with too much methodological details. Please rewrite and simplify.
  - Several missing or unclear points in the abstract have been revised for clarity and completeness.
- Some words and phrases are too technical for non-technical readers e.g. orthogonal fractional factorial design. While I understand that these terminologies represent underlying concepts, if possible, please simplify or explain further.
  - To enhance accessibility for non-specialist readers, several technical terms have been revised for clarity. These adjustments are reflected on page 6, lines 20 and 36; page 11, lines 7 and 19; and page 12, line 12.
- Results section focus too much on coefficients. A more narrative interpretation will make it clear for lay readers.
  - To enhance accessibility for non-specialist readers, several technical terms have been clarified. It was decided to report marginal willingness to pay values instead of beta coefficients. This change is reflected on page 10, line 14 of the revised manuscript.
- The discussion section should focus more on comparison with already available literature and public health policy implications of the research, rather than statistical details.
  - To address the reviewer's suggestion, we incorporated public health policy implications throughout the Discussion section. These additions are marked in yellow for ease of reference.
- The overall language is good. However, some sentences are overly complex and could be simplified.
  - Throughout the manuscript, complex and lengthy sentences have been revised to improve clarity and readability for a broader audience.

---

## VERSION 2 - REVIEW

---

| | |
|---|---|
| **Reviewer** | **1** |
| **Name** | **Senanayake, Sameera** |

**Affiliation** **Queensland University of Technology, Duke-NUS Medical School**

**Date** **16-Oct-2025**

**COI**

---

I thank the authors for addressing my comments.

---

**Reviewer** **2**

**Name** **Shahzad, Muhammad**

**Affiliation** **Khyber Medical University**

**Date** **26-Oct-2025**

**COI**

---

The authors have addressed all the comments and therefore, the manuscript can be accepted for publication.
